# Downregulation of Methionine Cycle Genes *MAT1A* and *GNMT* Enriches Protein-Associated Translation Process and Worsens Hepatocellular Carcinoma Prognosis

**DOI:** 10.3390/ijms23010481

**Published:** 2022-01-01

**Authors:** Po-Ming Chen, Cheng-Hsueh Tsai, Chieh-Cheng Huang, Hau-Hsuan Hwang, Jian-Rong Li, Chun-Chi Liu, Hsin-An Ko, En-Pei Isabel Chiang

**Affiliations:** 1 Department of Food Science and Biotechnology, National Chung Hsing University, Taichung 40402, Taiwan; yaoming9@yahoo.com.tw (P.-M.C.); edison881@gmail.com (C.-H.T.); khsinan@gmail.com (H.-A.K.); 2 Innovation and Development Center of Sustainable Agriculture (IDCSA), National Chung Hsing University, Taichung 40402, Taiwan; 3 Department of Life Science, National Chung Hsing University, Taichung 40402, Taiwan; cchuang@dragon.nchu.edu.tw (C.-C.H.); hauhsuan@dragon.nchu.edu.tw (H.-H.H.); 4 Institute of Genomics and Bioinformatics, National Chung Hsing University, Taichung 40402, Taiwan; fanicesiza@gmail.com (J.-R.L.); chunchiliu@gmail.com (C.-C.L.); 5 Early Diagnostics, The UCLA Magnify Incubator, Los Angeles, CA 90095, USA

**Keywords:** human hepatocellular carcinoma, MAT1A, GNMT, methionine cycle

## Abstract

The major biological methyl donor, *S*-adenosylmethionine (adoMet) synthesis occurs mainly in the liver. Methionine adenosyltransferase 1A (MAT1A) and glycine N-methyltransferase (GNMT) are two key enzymes involved in the functional implications of that variation. We collected 42 RNA-seq data from paired hepatocellular carcinoma (HCC) and its adjacent normal liver tissue from the Cancer Genome Atlas (TCGA). There was no mutation found in *MAT1A* or *GNMT* RNA in the 42 HCC patients. The 11,799 genes were annotated in the RNA-Seq data, and their expression levels were used to investigate the phenotypes of low *MAT1A* and low *GNMT* by Gene Set Enrichment Analysis (GSEA). The REACTOME_TRANSLATION gene set was enriched and visualized in a heatmap along with corresponding differences in gene expression between low *MAT1A* versus high *MAT1A* and low *GNMT* versus high *GNMT*. We identified 43 genes of the REACTOME_TRANSLATION gene set that are powerful prognosis factors in HCC. The significantly predicted genes were referred into eukaryotic translation initiation (EIF3B, EIF3K), eukaryotic translation elongation (EEF1D), and ribosomal proteins (RPs). Cell models expressing various *MAT1A* and *GNMT* proved that simultaneous restoring the expression of *MAT1A* and *GNMT* decreased cell proliferation, invasion, as well as the REACTOME_TRANSLATION gene *EEF1D*, consistent with a better prognosis in human HCC. We demonstrated new findings that downregulation or defect in *MAT1A* and *GNMT* genes can enrich the protein-associated translation process that may account for poor HCC prognosis. This is the first study demonstrated that MAT1A and GNMT, the 2 key enzymes involved in methionine cycle, could attenuate the function of ribosome translation. We propose a potential novel mechanism by which the diminished GNMT and MAT1A expression may confer poor prognosis for HCC.

## 1. Introduction

Hepatocellular carcinoma (HCC) is the most frequent primary liver cancer, and incidence of about 10.1 cases per 100,000 persons, and HCC is ranked as the sixth most common neoplasm and the third leading cause of cancer death [1]. HCC occurs mainly in the context of cirrhosis, hepatitis B or C virus infection, or nonalcoholic steatohepatitis, and its incidence is expected to increase [2]. Perturbations in folate dependent methylation pathways have been associated with cancer occurrence [3] and many human pathological conditions [4,5]. Genes involved in the folate-mediated one carbon (1C) metabolism have been important therapeutic targets for numerous human diseases [6,7,8,9] including HCC [10,11]. Enzymes of methionine cycle including methionine adenosyltransferases (MATs) and glycine N-methyltransferase (GNMT), are essential for the synthesis and utilization of *S*-adenosylmethionine (adoMet), the universal methyl donor and precursor for polyamine and glutathione synthesis [12,13,14].

*MAT* and *GNMT* genes are commonly diminished in human HCC and hepatoma cell lines [15,16,17,18,19]. *MAT1A* or *GNMT* dysregulation contributes to HCC progression; spontaneous HCC has been observed in the *mat1a*-knockout as well as in the *gnmt*-knockout mice [20,21,22]. Deletion of *gnmt* promotes the susceptibility to liver cancer in mice [22]; *gnmt* knockout mice exhibited elevated hepatic adoMet levels and *S*-adenosylhomocysteine hydrolase (SAHH) expression in the liver [22]. These studies suggested that defective MAT and/or GNMT proteins could be early markers in human HCC development.

Pleiotropic effect of MATs has been associated with global DNA hypomethylation and liver cancer progression and prognosis [16]. Mat1a knockout mice are predisposed to liver injury and hepatocarcinogenesis that displays increased proliferation [23].

Mammals have three distinct forms of MAT (MATI, MATII and MATIII), encoded by two distinct genes (*MAT1A* and *MAT2A*). MATs in the liver and in extrahepatic tissues are products of two genes, MAT1A and MAT2A, respectively. MATII consists of α2 catalytic subunit (encoded by *MAT2A*) and β regulatory subunit (encoded by *MAT2B)*. We recently discovered that *MAT1A* and *GNMT* were mostly expressed in the cytoplasm, whereas MAT2A showed both cytoplasmic and nuclear immunoreactivity, and that a higher cytoplasmic/nuclear (C/N) MAT2A expression ratio is correlated with poor overall survival in breast cancer patients [24].

HCC is characterized by the low expression of the liver-specific *MAT1A* gene that encodes the MATI/III isozymes; and the high expression of *MAT2A* that encodes the MATII isozyme, as well as high expression of *MAT2B* that encodes a β-subunit without catalytic action but can regulate MATII enzymatic activity [23].

We have demonstrated that, in the *GNMT* diminished HCC cell-line HepG2, restoration of *GNMT* assisted methylfolate-dependent homocysteine remethylation [25]. In *gnmt* transgenic and knockout mouse models, we discovered that *gnmt* expression can improve folate retention and bioavailability in the liver [25], decrease antifolate drug toxicity [25], improve DNA integrity, and reduce uracil misincorporation in the DNA [26,27]. We also demonstrated that *GNMT* expression enhances homocysteine transsulfuration and remethylation fluxes when methionine is in excess, and GNMT assists homocysteine clearance when needed [28].

Mechanisms of HCC inhibition by GNMT include: the suppression of dep domain-containing mTOR-interacting protein (DEPTOR) to the activation of mTOR targets SK6 and 4E-BP, that further impedes PI3K/AKT signaling pathway, the repression of the proteasomal degradation of phosphatidylinositol 3,4,5-trisphosphate-dependent Rac exchanger 2 (PREX2) by the E3 ubiquitin ligase HectH9, the maintenance of adequate proteins levels that related in the anti-oxidation and detoxification response and 1C metabolism pathways that could impede HCC development [28]. In *gnmt*-KO mice developed steatosis, fibrosis, and HCC, the methylation of RASSF1 and SOCS2 promoters and H3K27, which may result in epigenetic modulation of critical HCC carcinogenic pathways [21]. The above series of studies showed the essential and complex role of MAT1A and GNMT on maintaining optimal adoMet homeostasis, methylation balance, DNA integrity, and HCC prevention. In the present study we explored novel role of defected MAT1A and GNMT on HCC by curating gene sets from online pathway databases, publications in PubMed, and knowledge of domain experts by gene set enrichment analysis (GSEA) for profiling the effects of MAT1A and GNMT. We included 42 RNA-Seq data of matched HCC and adjacent normal liver tissues from TCGA and searched for MAT1A and GNMT-mediated novel biological processes/metabolic pathways by GSEA, and further investigated whether the newly identified genes could be involved HCC occurrence and development.

## 2. Results

### 2.1. HCC Patients with Low MAT1A and Low GNMT Expressions Had Poor Survival

**Figure 1:**
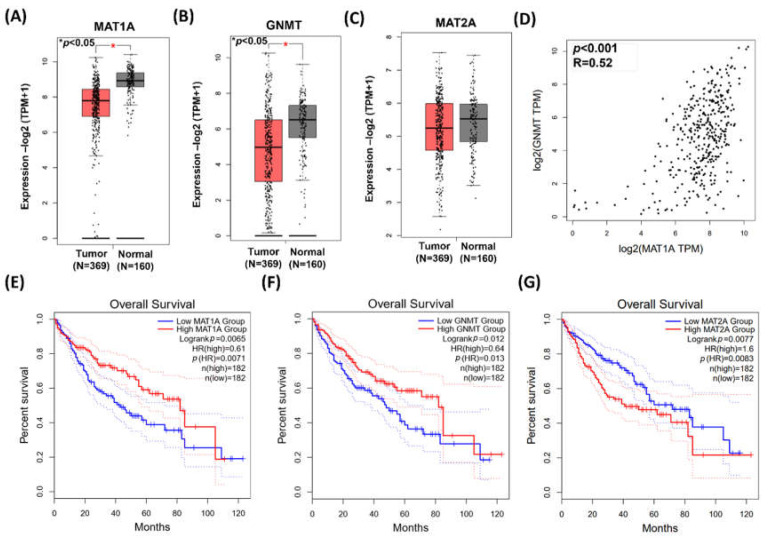
The expressions of *MAT1A*, *GNMT*, and *MAT2A* in HCC. Gene expression profiling interactive analysis (GEPIA) GEPIA2 database was employed for bioinformatics analysis. (**A**–**C**) *MAT1A*, *GNMT*, and *MAT2A* expression in paired tumor and adjacent normal tissue. *, *p* < 0.05. (**D**) *Pearson’s* correlation was used to elucidate *MAT1A* expression in relation to *GNMT* expression. (**E**,**F**) Kaplan–Meier plots showed that high expression of *MAT1A* and GNMT (**F**) are both associated with better overall survival rate of patients with HCC. HR, hazard ratio (**G**) Kaplan–Meier plot showed that elevated *MAT2A* expression is associated poor overall survival rate of patients with HCC. HR, hazard ratio.

Data from GEPIA demonstrated *MAT1A* and *GNMT* expressions were significantly higher in the adjacent normal tissues (*n* = 160) than those in the HCC tumor tissues (*n* = 369) (*p* < 0.001, Figure 1A,B). On the other hand, there was no statistical significance found in *MAT2A* between the adjacent normal and the HCC tumor tissues (Figure 1C). The gene expression of *MAT1A* and *GNMT* was highly correlated (*Pearson’s* correlation, *p* < 0.001, R = 0.52; Figure 1D). Kaplan–Meier plot (http://gepia2.cancer-pku.cn/#index accessed on 1 December 2020) showed that HCC patients with low *MAT1A* and low *GNMT* expression had poor survival rate (*p* = 0.0071 and *p* = 0.013, respectively, Figure 1E,F). In contrast, the survival curves indicated that high expression of *MAT2A* is associated with poor overall survival (Figure 1G).

### 2.2. HCC RNA-seq Data Collected and Analysis from TCGA Database

**Figure 2:**
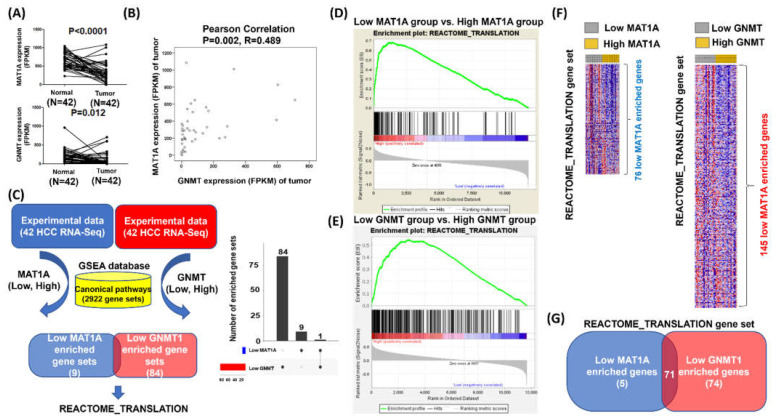
Searching for the potential biological processes associated with low expression of *MAT1A* and *GNMT*. (**A**) *MAT1A* and *GNMT* expressions in paired tumor and adjacent normal tissues in the HCC cases (*n* = 42). (**B**) Pearson’s correlation for *MAT1A* and *GNMT* expression in the 42 HCC cases. (**C**) Flow chart for searching the candidate biological processes that could be driven by low expression of *MAT1A* and *GNMT*; GSEA was used to process the 42 HCC RNA-seq data. REACTOME_TRANSLATION gene set was found to be significantly enriched in HCC with low *MAT1A.* The median of mRNA expression level was used as the cutoff point for the dichotomization of MAT1A and GNMT. A score greater than median was defined as “high” expression, whereas a score of less than or equal to median was defined as “low” expression, and the categories further were mapped into GSEA databases. (**D**) and in HCC with low *GNMT* (**E**). (**F**) Heatmaps of the REACTOME_ TRANSLATION enrichment genes in HCC with high and low MAT1A and GNMT categories (**G**) A total of 71 overlapped genes of the REACTOME_ TRANSLATION gene set that are enriched in both low *MAT1A* and low *GNMT* HCC samples.

Based on the above data, we then explored the data from 42 paired HCC tumor and adjacent normal tissues collected from the Cancer RNA-Seq Nexus [29], and identified the enriched pathways associated with defected *MAT1A* and *GNMT* expression. Low expression of *MAT1A* and *GNMT1* were confirmed in the tumor tissues as compared to those in the adjacent normal tissues (for *MAT1A* and *GNMT*, *p* < 0.0001 and *p* = 0.012, respectively (Figure 2A), consistent with the results obtained from the web server. *Pearson’s* correlation also revealed a statistically significant correlation between *MAT1A* and *GNMT* expression (R= 0.489, *p* = 0.002, Figure 2B).

Gene set enrichment analysis (GSEA) was then performed to explore novel function of *MAT1A* and *GNMT*, in which the gene expression level greater than the median was defined as “high”, whereas less than the median was defined as “low” (Figure 2C). The low *MAT1A* category has 10 gene sets enriched as compared with the high *MAT1A* category; the low *GNMT* category has 85 gene sets enriched compared with the high *GNMT* category from GSEA (Figure 2C). The Venn plots revealed that the REACTOME_TRANSLATION gene set was enriched in both categories (Figure 2C, left panel is Venn diagram, and right is Venn bar). The statistical significance (nominal *p* value) of the ES for the two categories was calculated using an empirical phenotype-based permutation test procedure that preserves the complex correlation structure of the gene expression data for *MAT1A* (Figure 2D) and *GNMT* (Figure 2E). Heatmaps of the REACTOME_TRANSLATION genes were made in low and high expressions of *MAT1A* and GNMT (Figure 2F). Further overlapping analyses of low *MAT1A* (versus high *MAT1A*) expression and low *GNMT* (versus high *GNMT*) expression revealed 71 genes involved the false discovery rate (FDR) against a chance finding at the typical threshold of 0.05 (Figure 2G). The Venn diagram in Figure 2G summarizes the intersection of 71 genes identified from low *MAT1A* (versus high *MAT1A*) expression and low *GNMT* (versus high *GNMT*) expression.

### 2.3. Forty-Three REACTOME_TRANSLATION Genes Selected from Low Expressions of GNMT and MAT1A Are Associated with Poor HCC Prognosis

**Figure 3:**
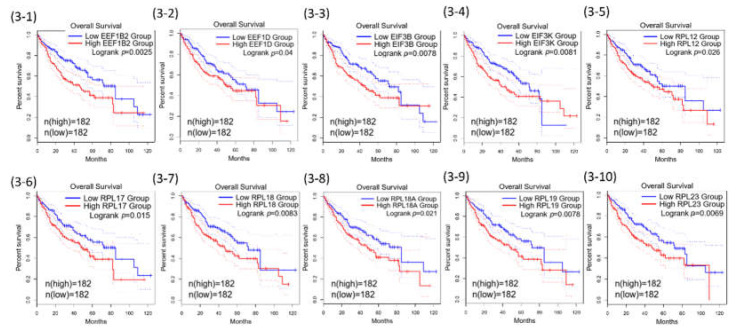

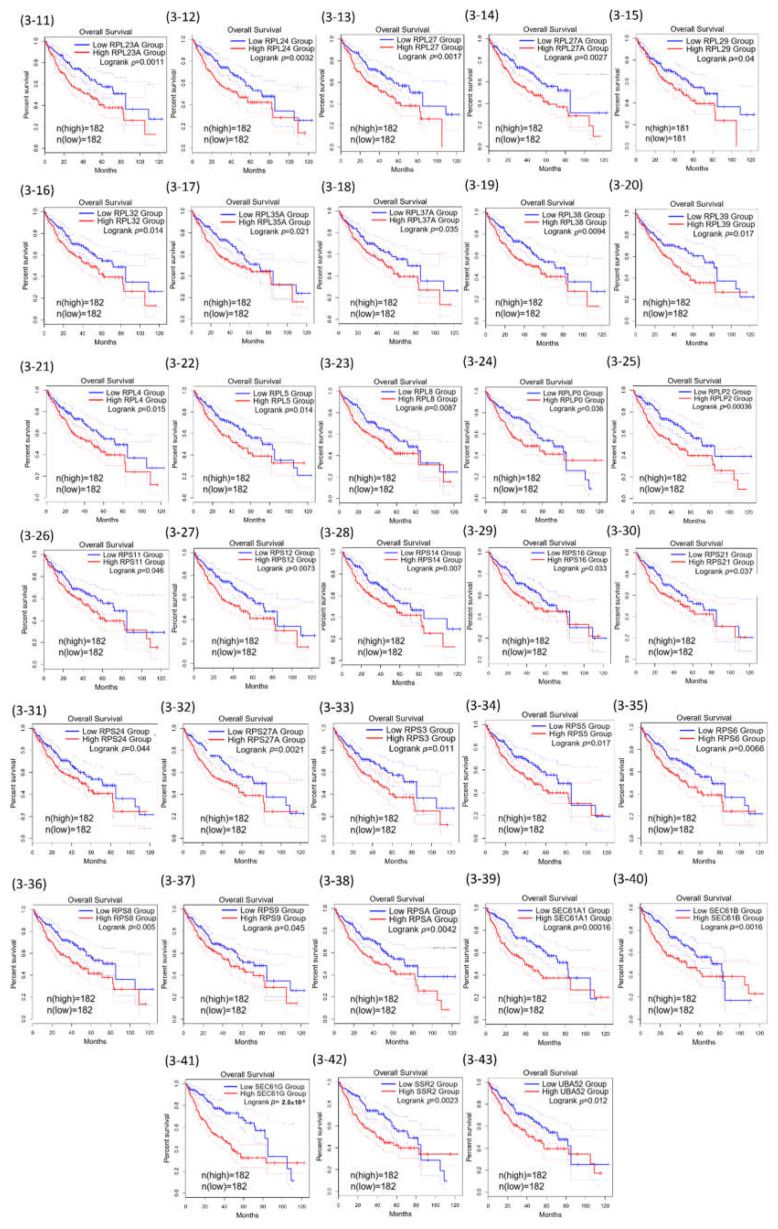
Kaplan–Meier curves for the 43 of REACTOME_TRANSLATION genes.

Among the 71 common genes in the REACTOME_ TRANSLATION gene set, further prognostic analysis demonstrated that 43 out of the 71 were statistically associated with poor overall survival of HCC (Table 1). The 43 Kaplan–Meier survival plots were performed after the samples were classified into high- and low-expression groups according to the median scores (Figure 3).

Among these targets, eukaryotic translation elongation factor 1 delta (EEF1D) has been reported to modulate proliferation and epithelial-mesenchymal transition in oral squamous cell carcinoma [30] and promote glioma proliferation, migration, and invasion through epithelial-mesenchymal transition and PI3K/Akt pathway [31]. For eukaryotic translation initiation factor 3, subunit B (EIF3B) expression was found to be upregulated in gastric cancer tissues; it is strongly associated with proliferating cell nuclear antigen (PCNA) expression and is associated with poor outcomes in gastric cancer patients [32]. These studies suggest that EEF1D and EIF3B may play an oncogenic role in human cancer progression and whether they can serve as independent prognostic factors for HCC patients was further investigated in our cell models.

### 2.4. Expression of MAT1A and GNMT Decreased Cell Proliferation, Invasion

**Figure 4:**
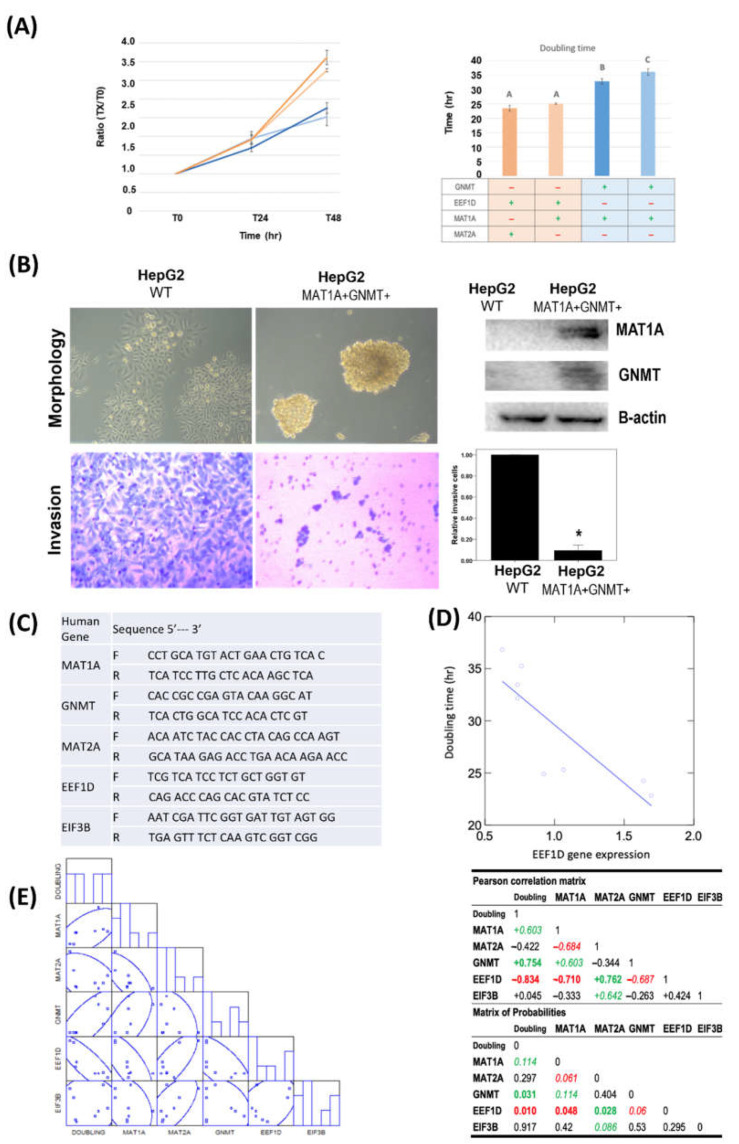
*MAT1A* and *GNMT* expression decreased cell proliferation and invasion in HepG2 cells. *MAT1A* and *GNMT* expression also decreased reactome protein *EEF1D*, and *EIF3B* mRNA levels. (**A**) Doubling time of 4 HepG2 derived stable cell lines with various *MAT1A* and *GNMT*-expression levels were determined by cell counting. Points, mean; bars, SE. A: Control cells. B: Clone #1, *MAT1A* and *GNMT*-overexpressing cells. C: Clone #2, *MAT1A* and *GNMT*-overexpressing cells. (**B**) Overexpression of MAT1A and GNMT decreases invasive ability of HCC cells. MAT1A, GNMT, and β-actin (loading control) protein expression levels were evaluated by immunoblotting in HepG2 and HepG2/MAT1A/GNMT. *, *p* < 0.05. (**C**) List of quantitative PCR primers. (**D**) Pearson’s correlation matrix of *GNMT*, *MAT1A*, *MAT2A*, *EEF1D*, and *EIF3*B mRNA expression. (**E**) Linear plot of correlation estimated from doubling time and *EEF1D* mRNA expression.

To investigate the impacts of *MAT1A* and *GNMT* expression on human hepatoma cells, we utilized cell models that expressed various levels of these 2 genes [33]. The cell numbers of wildtype HepG2 (WT) and HepG2 derived *MAT1A* and *GNMT*-expressing cell-lines (MAT1A+GNMT+) are shown in Figure 4A. Wild-type HepG2 grew faster than the cell-line stably transfected with *MAT1A* and *GNMT (MAT1A+GNMT+)*; *MAT1A+GNMT+* cells exhibited decreased cell growth (Figure 4A). The results of cell proliferation inspired us to further investigate the impacts of *MAT1A* and *GNMT* expression on cell invasion ability. Boyden chamber assay revealed that expressing *MAT1A* and *GNMT* in cells diminished with both genes significantly reduced the invasion ability of HepG2 cells (Figure 4B).

Since the expression of *MAT1A* and *GNMT* in HepG2 cells appeared to impede HCC cell proliferation, we further investigated the correlations between the cell doubling time and the expression levels of *MAT1A*, *MAT2A*, *GNMT*, as well as selected REACTOME gene EEF1D and EIF3B by quantitative real-time PCR using designated primers (Figure 4C). *MAT1A* and *GNMT* expression levels both positively correlated with cell doubling time (Pearson’s correlation for *MAT1A*: R= 0.603, *p* = 0.114; *GNMT*: R= 0.754, *p* = 0.031, Figure 4D) whereas no correlation was found between doubling time and *MAT2A*. *EEF1D* inversely correlated with *MAT1A* (R= −0.710, *p* = 0.048) and *GNMT* (R= −0.687, *p* = 0.06). In contrast to *MAT1A* and *GNMT* that were inversely correlated with *EEF1D*, *MAT2A*, a gene frequently highly expressed in HCC, was positively correlated with *EEF1D* (R= 0.762, *p* = 0.028) (Figure 4E). *EEF1D* expression was found to have a strongly inverse correlation with cell doubling time (R= −0.834, *p* = 0.010) (Figure 4E). These results indicated that *EEF1D* may play a significant role in HCC progression independent of protein translation, and *MAT1A*, *GNMT* and *MAT2A* may modulate HCC proliferation/and or progression through the expression of REATOME gene *EEF1D.*

### 2.5. MAT1A and GNMT Are Negatively Correlatived with EEF1D and EIF3B in

**Figure 5:**
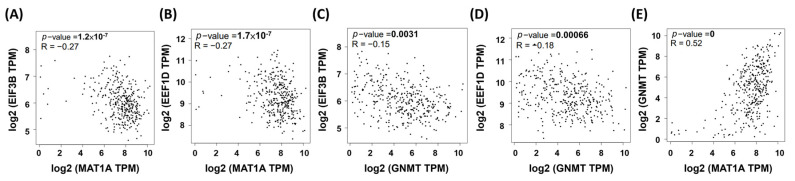
The correlations between MAT1A and GNMT with reactome genes in liver hepatocellular carcinoma patients using the Cancer Genome Atlas (TCGA) web server program. Pearson’s correlations showed that MAT1A mRNA levels inversely correlated with (**A**) EIF3B and (**B**) EEF1D; GNMT mRNA levels inversely correlated with EIF3B (**C**) and EEF1D (**D**); MAT1A (**E**) and GNMT expressions are positively correlated.

Using GSEA to process the 42 HCC RNA-seq data, we identified overlapped genes of the REACTOME_ TRANSLATION gene set that are enriched in both low *MAT1A* and low *GNMT* HCC samples. In our cell models, *MAT1A* and *GNMT* expression significantly decreased cell proliferation and invasion in HepG2 cells. In particular, *EEF1D* expression was inversely correlated with *MAT1A* and *GNMT* expression, as well as with cell doubling time. HCC RNA-seq data were then collected from The Cancer Genome Atlas (TCGA) datasets and analyzed by Gene Expression Profiling Interactive Analysis (GEPIA) [34]. Correlation of *EEF1D*, *EIF3B*, *MAT1A*, and *GNMT* mRNA expression were performed using GEPIA plotters (Figure 5). These results indicated that our approaches searching for the potential biological processes associated with low expression of *MAT1A* and *GNMT* were affective.

## 3. Discussion

In the present study, bioinformatic analysis based on TCGA HCC database revealed the significant associations between the expression level of REACTOME_TRANSLATION genes and poor prognosis of HCC. These results raised the possibility that low expression of *MAT1A* and *GNMT* confers poor prognosis of HCC patients via REACTOME_ TRANSLATION biological process.

To further explore the biological relevance of this differential expression pattern, gene-set enrichment analyses (GSEA) were performed in the RNA-seq data from 42 paired HCC patients. The heatmaps and the Venn diagram revealed that 43 of the 71 REACTOME_TRANSLATION genes (60.1%) overlapping with low expressions of *MAT1A* and *GNMT* are associated with poor prognosis in HCC, indicating that REACTOME_TRANSLATION could potentially participate in human HCC tumorigenesis.

Transcriptional dysregulation has been recognized as a hallmark in cancer development, but relatively less is known about the dysregulation of gene expression at the translational level [35]. Translation and translational control are critical in stress adaptation of cancer cells to overcome challenges from the tumor microenvironment, immune recognition, their own continuous replication, and therapeutic modalities. Therefore, changes in the translational machinery can mediate the oncogenic signaling [35].

### 3.1. Ribosomal Proteins

Our approach in combination with Kaplan–Meier survival plot revealed numerous ribosomal protein (RP) encoding genes that were statistically associated with poor overall survival of HCC. Ribosome is the essential cellular organelle for protein synthesis that consists of ribosomal RNAs (rRNAs) and RPs. Human ribosomes are made up of four rRNAs species and about 80 different RPs. RP genes encode for ribosomal proteins that make up the ribosomal subunits involved in the cellular process of translation. Many RPs also play various roles independent of protein biosynthesis.

Some extra-ribosomal functions of ribosomal proteins have been reported in human colorectal cancers previously. Among the RPs that were found to be associated with poor overall HCC survival in our present study, RPL18 (Figure 3-7) and RPS27 (Figure 3-13) have been reported in cell growth or proliferation regulation; RPL23 (Figure 3-10) and RPL5 (Figure 3-22) have been reported in tumor suppressor gene regulation; RPL35A (Figure 3-17) has been reported in cell apoptosis regulation; RPLP0 (Figure 3-24) was reported to be involved in DNA repair; RPS14 (Figure 3-29) and RPS8 (Figure 3-36) have been reported in self-translation regulation in many human cancers, HCC included [36,37,38]. Other RPs including RPL19, RPL4, RPLP2, RPS10, RPS12, RPS3, RPS5, RPS6, RPS9 have been reported to participate in self-translation regulation, RNA splicing and modification, transcription regulation, DNA repair, and developmental regulation in in other species [36,37,38]. The expression of RPL19 is associated with poor overall survival of HCC in the present study (Figure 3-9). The xenografted tumors with knocked-down RPL19 were significantly smaller than those in the control tumors, and siRNA knockdown of ribosomal protein gene RPL19 abrogates the aggressive phenotype of human prostate cancer [39,40,41]. These findings and the extra-ribosomal regulatory functions of RPL19 beyond protein synthesis provide a potential target for controlling the human HCC cellular phenotype.

### 3.2. ER Transmembrane Proteins and Ubiquitin Fusion Protein

Our study also revealed numerous genes encoding for the protein transport protein Sec61 subunits were statistically associated with poor overall survival of HCC, including SEC61A1, SEC61B, and SEC61G. The Sec61 complex is the central component of the protein translocation apparatus of the endoplasmic reticulum (ER) membrane. The Sec61 complex forms a transmembrane channel where proteins are translocated across and integrated into the ER membrane. Targeting human epidermal growth factor receptor 3 (HER3) by interfering with its Sec61-mediated co-translational insertion into the endoplasmic reticulum has been identified as a novel strategy to eliminate HER3 function [42]. Higher expression of SEC61G has been reported in HCC tissues than adjacent tissues. knockdown of SEC61G inhibited cell proliferation and induced cell apoptosis in vitro. SEC61G was required for cell migration and invasion, conferring a potential role for SEC61G in tumor transfer [43].

Ubiquitin A-52 residue ribosomal protein fusion product 1 (UBA52) gene that encodes for the 60S ribosomal protein L40 (RPL40) was statistically associated with poor overall survival of HCC in the present study. Ubiquitin fusion proteins have been overexpressed in colon cancer [44]. Degradation of CCNB1 mediated by APC11 through UBA52 ubiquitination promotes cell cycle progression and proliferation of non-small cell lung cancer cells [45].

### 3.3. Eukaryotic Translation Initiation and Elongation Factors

Our study revealed numerous genes encoding for the eukaryotic translation initiation (EIF3B, EIF3K) and elongation factors (EEF1B2, EEF1D) were statistically associated with poor overall survival of HCC. Eukaryotic translation initiation factor 3 (eIF3), the largest translation initiation factor composed of 13 non-identical polypeptides, plays an important role in protein synthesis that bridges the 43S preinitiation complex and eIF4F-bound mRNA [46]. The aberrant expression of eIF3 subunits was detected in various human cancers, and it was proposed to be a novel target in drug development [47]. Overexpression of the translation elongation factor 1 complex (eEF1) subunits was observed in 72% human cardioesophageal carcinoma [48]. Translation elongation factors have been proposed to play a role in tumorigenesis and affect survival in cancer specific manner [49]. EIF3B expression was upregulated in gastric cancer tissues and is associated with poor outcomes in gastric cancer patients [47]. EIF3B promoted gastric cancer cell proliferation, enhanced tumor cell migration and invasion through epithelial-mesenchymal transition (EMT) and the Stat3 signaling pathway in numerous human cancers [47]. Downregulation of EIF3B inhibited proliferation and metastasis of gastric cancer [50]. Knockdown of EIF3B in gastric cancer cells suppressed the growth of xenograft tumors and lung metastatic colonization in vivo [51]. The role of EIF3B in HCC remained to be determined.

In the present study, we demonstrated that important 1C metabolic gene *MAT1A* and *GNMT* are both strong prognostic indicators for HCC. Data from HCC patients with both genes diminished revealed that the REATOME pathway could participate in HCC tumorigenesis. Using cell models with various level of MAT1A and GNMT, we further identified a REATOME gene *EEF1D*; its expression level is not only inversely related to both genes but also is closed related to cell proliferation.

MAT1A and GNMT are both critical regulators for *S*-adenosylmethionine homeostasis of (adoMet) and methylation status [27,28]. Perturbations in folate dependent methylation pathways have been associated with increased human cancer risk. Lower serum folate has been shown to be associated poor survival of gastric cancer patients [52]. GNMT is a folate binding protein that can promote methylene-folate dependent pyrimidine and formyl-folate dependent purine synthesis in HCC [27]. We have demonstrated that GNMT facilitates the conservation of methyl groups by limiting homocysteine remethylation fluxes, controls transmethylation kinetics and S-adenosylmethionine (adoMet) homeostasis [28]. Restoring GNMT assists methylfolate-dependent reactions and ameliorates the consequences of folate depletion. GNMT expression in vivo improves folate retention and bioavailability in the liver [25]. Loss of GNMT impairs nucleotide biosynthesis; restoring GNMT expression enhances nucleotide biosynthesis and improves DNA integrity by reducing uracil misincorporation in DNA both in vitro and in vivo [26]. GNMT therefore has a protective role in cellular defense against DNA damage and human cancer [26].

**Figure 6:**
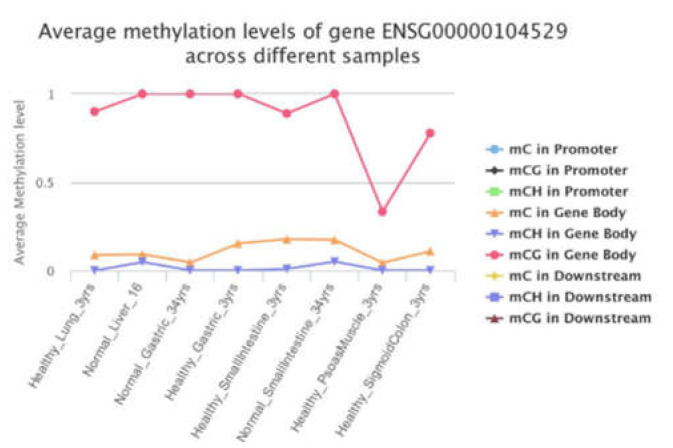
Promoter and gene body methylation status of *EEF1D*.

The present study indicated that restoring *MAT1A* and *GNMT* expression may suppress EEF1D expression that is potentially oncogenic in human cancer progression. Dynamic alteration of the epigenome are important regulatory processes in a biological function. Cancer is a multifactorial disease characterized by aberrant epigenetic controls. The significant correlations among *EEF1D* and *MAT1A*, *GNMT*, and *MAT2A* in our cell models raise the possibility that its expression might be controlled by 1C metabolism mediated epigenetic modification. DNA methylation levels of *EEF1D*’s first CpG island have been reported to be negatively correlated with its gene expression levels in cattle [53], supporting our postulation that one carbon metabolism may control gene expression of *EEF1D* via modulation of methylation. We examined promoter and gene body methylation status of EEF1D using the methylation bank (MethBank http://bigd.big.ac.cn/methbank/ accessed on 1 October 2021). MethBank is a comprehensive methylation database that features consensus reference methylomes (CRMs), single-base resolution methylomes (SRMs), single-cell methylation maps and open platform for epigenome-wide association studies and integrates DNA & RNA methylation tools. Complete mCG methylation was shown in the gene body of *EEF1D* in normal healthy liver (Figure 6).

Whether EEF1D methylation status is altered in human HCC, and whether MAT1A and GNMT functions affect EEF1D expression or other Reactome pathway genes, remains to be determined. Methylation profiling in cells with various *MAT1A* and *GNMT* expression are underway.

## 4. Materials and Methods

### 4.1. Data Collection

We collected a total of 42 of paired tumor and tumor-adjacent normal HCC tissue RNA-Seq datasets from the Cancer RNA-Seq Nexus (CRN, http://syslab4.nchu.edu.tw/CRN accessed on 1 October 2019) that has a user-friendly web interface designed to facilitate cancer research and personalized medicine [29,54].

### 4.2. Gene Set Enrichment Analysis

The median of mRNA expression was used as the cutoff point for the dichotomization of MAT1A and GNMT. A score greater than median was defined as “high” expression, whereas a score of less than or equal to median was defined as “low” expression, and the categories further were mapped into GSEA databases. This was performed using java GSEA Desktop Application from the Broad Institute at MIT. The MSigDB gene sets are divided into eight major collections: hallmark gene sets are coherently expressed signatures derived by aggregating many MSigDB gene sets to represent well-defined biological states or processes (H), positional gene sets for each human chromosome and cytogenetic band (C1), curated gene sets from online pathway databases, publications in PubMed, and knowledge of domain experts (C2), regulatory target gene sets based on gene target predictions for microRNA seed sequences and predicted transcription factor binding sites.(C3), computational gene sets defined by mining large collections of cancer-oriented microarray data (C4), GO gene sets consist of genes annotated by the same GO terms (C5), oncogenic gene sets defined directly from microarray gene expression data from cancer gene perturbations (C6), and immunologic gene sets defined directly from microarray gene expression data from immunologic studies (C7). Our data set had 11,799 genes, and the collections cp (canonical pathways: Biocarta, KEGG, and Reactome, total 2922 gene sets) was used for mapping by running GSEA (https://www.gsea-msigdb.org/gsea/index.jsp, accessed on 1 December 2020).

### 4.3. Survival Analysis 25

GEPIA2 performs survival analyses based on gene or isoform expression levels [34]. Given a list of custom cancer types, GEPIA2 would provide a heat map and show the survival analysis based on multiple cancer types [34]. The red blocks denote the higher and blue ones the lower risk, with an increase in the gene or isoform expression. The blocks with darkened frames indicate statistical significance in prognostic analyses. Since gene survival analyses can be context-dependent, this function allows users to screen for the prognostic impact of a gene or an isoform across different cancer types (http://gepia2.cancer-pku.cn/#index, accessed on 1 October 2021).

### 4.4. Cell Models with and without GNMT and MAT1A Expression

To elucidate how *MAT1A* and *GNMT* may affect cell proliferation, invasion, and Reactome pathway genes, stable cell-lines established from the human hepatoma cell-line HepG2 were used. One stable cell-line was established by cotransfecting pGNMT and pTK-Hyg plasmid (Clontech, Takara Bio USA, Inc., San Jose, CA, USA) DNAs that represented a model with normal GNMT function (GNMT+). The negative control cell-line, GNMT- was established by stable transfection with pFLAG-CMV-5. Establishment of stable cell-lines was described in detail previously and GNMT expression was confirmed by Western blot analyses [25]. Human *MAT1A* cDNA clone pCMVSPORT6 was obtained from National Yang-Ming University VYM Genome research center (Taipei, Taiwan, ROC). The full-length cDNA clone of human MAT1A was cloned into the mammalian expression vector pcDNA3. Human hepatoma cell line HepG2 cells expressing *GNMT* were further transfected with pcDNA3-MAT1a and underwent G418 (Sigma-Aldrich, Inc., St. Louis, MO, USA) selection. Multiple clones were selected, and *MAT1A* and *GNMT* expression was analyzed by real-time PCR (Applied Biosystems; Thermo Fisher Scientific, Inc., Waltham, MA, USA). Detailed procedures on the establishment of cell models and the characteristics of these cell-lines will be submitted elsewhere (Ko et al. manuscript in preparation).

### 4.5. Cell Proliferation in HCC Cell-Lines Expressing Various MAT1a and GNMT

All chemicals were purchased from Sigma-Aldrich, Inc. via the local distributor in Taiwan unless otherwise specified. Wildtype HepG2 and HepG2 derived cell-lines with various *MAT1A* and *GNMT* expressions were cultured in αminimum essential medium α (αMEM) with 10% fetal bovine serum (FBS), 1% Penicillin-Streptomycin-Amphotericin B Solution (PSA), and cultured at 37 °C in a 5% CO_2_ incubator. The cell growth and doubling time were compared between wildtype HepG2 and other cell-lines expressing *MAT1A* and *GNMT*.

### 4.6. Correlations between GNMT, MAT1A, and MAT2A and REACTOME Genes in Human HCC Cell-Lines

Total RNA was isolated by trizol and RNA integrity was checked by electrophoresis. RNA was converted to cDNA. Gene expression was determined by quantitative real-time PCR (ABI7000, Applied Biosystems; Thermo Fisher Scientific, Inc., Waltham, MA, USA). using Sybr green. Primers used for target genes are shown in Figure 4B. The expression of each gene was calculated by normalizing the threshold cycle value of target gene to that of the control housekeeping gene 18sRNA [55].

### 4.7. Transwell Invasion Assay

Cell invasion was investigated using Matrigel invasion chambers with a pore size of 8 μm (Costar; Corning Life Sciences, Cambridge, MA, USA) as described [56]. Briefly, wildtype HepG2 and stable cell-lines expressing various levels of MAT1A and GNMT (4 × 10^4^ cells per chamber) in serum-free medium were seeded in the upper chamber, and 10% fetal bovine serum (FBS) (Gibco; Thermo Fisher Scientific, Inc., Waltham, MA, USA) was used as a chemoattractant in the bottom well. After incubation for 24 h at 37 °C, the non-invasive cells on the upper surface of the membrane were removed with a cotton swab, and the invasive cells on the bottom side were fixed in 100% methanol at room temperature for 5 min, stained with 1% crystal violet at room temperature for 10 min and counted using a microscope (Nikon Eclipse 80i; Nikon Corporation Tokyo, Japan) under ×200 magnification with three replicate wells of view per cells.

### 4.8. Western Blot Analysis

The cells were harvested using a curet and centrifuged at 1000× *g* for 10 min at 4 °C and then lysed in ice-cold radioimmunoprecipitation assay (RIPA) lysis buffer (Catalog number: 89900, Thermo Fisher Scientific, Inc., Waltham, MA, USA) with 100 µL protease inhibitor cocktail (Roche, San Francisco, CA, USA). Equal amounts of protein (30 µg) were separated by SDS-PAGE (10% gel) and subsequently transferred to a polyvinylidene difluoride membrane. Subsequent to blocking with 5% skimmed milk at room temper-ature for 1 h, the membranes were incubated at 4 °C overnight with primary antibodies, including anti-GNMT (1:1000; GTX64826; GeneTex, Irvine, CA, USA), anti-MAT1A (1:5000; GTX132095; GeneTex), anti-β-actin (1:1000; GTX629630; GeneTex), followed by incubation at room temperature for 2 h with HRP-conjugated polyclonal secondary antibody (1:5000; GTX213110-01/GTX213111-01; GeneTex) [11]. All Western blots were visualized using the enhanced plus chemiluminescence assay kit (EMD Millipore, Billerica, MA, USA), according to the manufacturer’s protocol. Protein expression levels in cells were quantified by ImageJ software (Analytik Jena US LLC, Upland, CA, USA, https://imagej.nih.gov/ij/, accessed on 11 November 2021).

### 4.9. Statistical Analysis

Pearson’s correlation was conducted using SYSTAT (SYSTAT Software Inc., Chicago, IL, USA) and SPSS (PASW Statistics 18.0, SPSS Inc., Chicago, IL, USA). Independent-sample *t*-test was respectively used for binary variables and continuous variables to compare liver tumor and their adjacent liver tissues. The *p*-value of the test was 2-tailed with a level of significance (α) = 0.05. A *p*-value of less than 0.05 indicated statistical significance.

## 5. Conclusions

Dynamic alteration of the epigenome are important regulatory mechanisms in biological function. Our study indicated that restoring *MAT1A* and *GNMT* expression may suppress EEF1D expression that is potentially oncogenic in human cancer progression. This is the first study demonstrated that MAT1A and GNMT, the 2 key enzymes involved in methionine cycle, could potentially attenuate cancer progression via suppression of ribosome translation. More studies on methylation profiling in cells with various *MAT1A* and *GNMT* expression are warranted.

## Figures and Tables

**Table 1:** List of 71 overlapped genes in the REACTOME_TRANSLATION gene set that are enriched in both low *MAT1A* and low *GNMT* HCC samples.

EEF1B2 *	RPL13A	RPL27A *	RPL38 *	RPLP2 *	RPS2	RPS5 *	UBA52 *
EEF1D *	RPL17 *	RPL28	RPL39 *	RPS10	RPS21 *	RPS6 *	
EEF1G	RPL18 *	RPL29 *	RPL4 *	RPS11 *	RPS23	RPS8 *	
EIF3B *	RPL18A *	RPL31	RPL41	RPS12 *	RPS24 *	RPS9 *	
EIF3G	RPL19 *	RPL32 *	RPL5 *	RPS13	RPS27	RPSA *	
EIF3K *	RPL23 *	RPL34	RPL7	RPS14 *	RPS27A *	SEC61A1 *	
FAU	RPL23A *	RPL35	RPL7A	RPS15	RPS29	SEC61B *	
RPL10A	RPL24 *	RPL35A *	RPL8 *	RPS16 *	RPS3 *	SEC61G *	
RPL12 *	RPL26	RPL36	RPLP0 *	RPS18	RPS3A	SSR2	
RPL13	RPL27 *	RPL37A *	RPLP1	RPS19	RPS4X	SSR4	

* Statistical significance for prognosis genes using GEPIA.

## Data Availability

The data presented in this study are available on request from the corresponding author.
